# Lives in a gram of soil: linking life cycle biology to soil ecology

**DOI:** 10.1093/ismejo/wrag215

**Published:** 2026-08-20

**Authors:** Jordi van Gestel

**Affiliations:** Developmental Biology Unit, European Molecular Biology Laboratory, Meyerhofstraße 1, Heidelberg 69117, Germany; Molecular Systems Biology Unit, European Molecular Biology Laboratory, Meyerhofstraße 1, Heidelberg 69117, Germany

**Keywords:** life cycle biology, life cycle ecology, soil ecology, soil communities, microbial diversity

## Abstract

Soils are among the most diverse but least understood environments on the planet. Just below our feet, secluded from our sight, we find complex communities of bacteria, fungi, protists, and animals. These communities organize themselves in both time and space, from fast to slow growers, from decomposers to predators, from early colonizers to late arrivers. Despite a growing understanding of community dynamics, the lives of organisms within the soil often remain poorly understood: how do organisms grow, propagate, and disperse within their particle-packed environments? In this essay, I reflect on the microbial life cycles found in soils. Starting from a single gram of soil collected on our campus, I explore the conceptual building blocks underlying life cycle biology, how life cycles emerge within their environment, and how they are coupled between species. Microbes express widely diverse life cycle motifs with many unique life stages promoting growth and/or propagation. These life stages have a major impact on community ecology; they determine the fate of microbes within soils and affect how, when, and where microbes interact. A life cycle perspective is thus essential for deepening our understanding of the community dynamics that unfold underfoot.

Soils are fundamental to life on Earth; they drive nutrient cycling, support above-ground communities, and promote ecological succession. Despite an increasing societal awareness of the essential importance of soils for both man-made and natural environments [1, 2], they remain one of the least understood environments on the planet, which relates to their complex biology. A single gram of soil easily contains millions of bacterial cells, meters of fungal hyphae, and tens of thousands of micro- and mesoscopic predators, including an abundance of protists and nematodes [3–9]. Observing these organisms in the opaque soil environment is difficult. Even when some of them are spotted through microscopy, identifying species is problematic, and following individuals over time is practically impossible.

Sequencing-based approaches have provided a powerful way out. By amplicon sequencing and metagenomics, soil diversity can nowadays be mapped out on a global scale [10]. These efforts revealed a core set of mostly microbial taxa that are repeatedly found in soil communities [11]. These taxa show consistent differences in abundance across soil environments, depending on the abiotic conditions (e.g. porosity, acidity, and precipitation) [10, 12–14], and undergo reproducible changes in abundance following small- or large-scale environmental perturbations [15], from the rapid community dynamics after rainfall [16] to the more gradual changes across seasons [17, 18].

Although sequencing methods have uncovered much of the unseen soil diversity, they did not replace the need for observations. Microbial life cycles emerge from interactions between microbes and their environment, and only by monitoring microbes in both time and space can we understand the functional implications of their behaviors: how do microbial behaviors affect growth, propagation, and dispersal in soil environments? To complement sequencing-based approaches, an increasing number of studies therefore examine soil communities through observational assays in experimental setups that mimic the soil: microfluidic environments make it possible to directly follow microbes in “soil-on-a-chip” experiments [19–26], synthetic soils reveal how microbes propagate in porous environments [25, 27–30], and high-content microscopy makes it possible to study how microbes behave across a broad range of biotic and abiotic conditions [31, 32]. These observational assays are furthermore boosted by developments in computer vision, making it possible to automatize image analysis at scale [33–37].

Motivated by these scientific advances, I will here reflect on microbial life cycles in soils (see fn. 1, Supplementary Text 1), highlighting their complexities and promise for deepening our understanding of soil communities. Starting from the diversity in a single gram of soil collected under a few deciduous beech trees on our campus in Heidelberg, I will briefly delve into the biotic and abiotic soil environment, lay out the conceptual building blocks needed to study microbial life cycles, and explore the coupling and coexistence of disparate life cycles. The link between life cycle biology and soil ecology provides exciting opportunities for future research on which I will reflect in the concluding paragraphs of this essay.

## In a gram of soil

To set the stage, I start from the taxonomic diversity within a gram of soil collected on our campus, which uncovers a small number of broader taxonomic groups dominating the **soil community** (see Glossary; Fig. 1): for prokaryotes, these include the *Proteobacteria*, *Actinobacteriota*, *Acidobacteriota*, *Planctomycetota*, *Verrucomicrobiota*, *Bacteroidota*, and *Chloroflexi*, and for eukaryotes the *Fungi*, *Cercozoa*, *Metazoa*, *Apicomplexa*, *Gyrista*, *Ciliophora*, and *Evosea*. These taxonomic groups are not unique to our soil sample, but ubiquitously observed across soils, suggestive of a core community of interacting taxa [5, 6, 11, 38]. The same pattern is repeated at lower taxonomic levels as well, where a small number of families dominates each of the broader taxonomic groups (Fig. 1).

**Figure 1:**
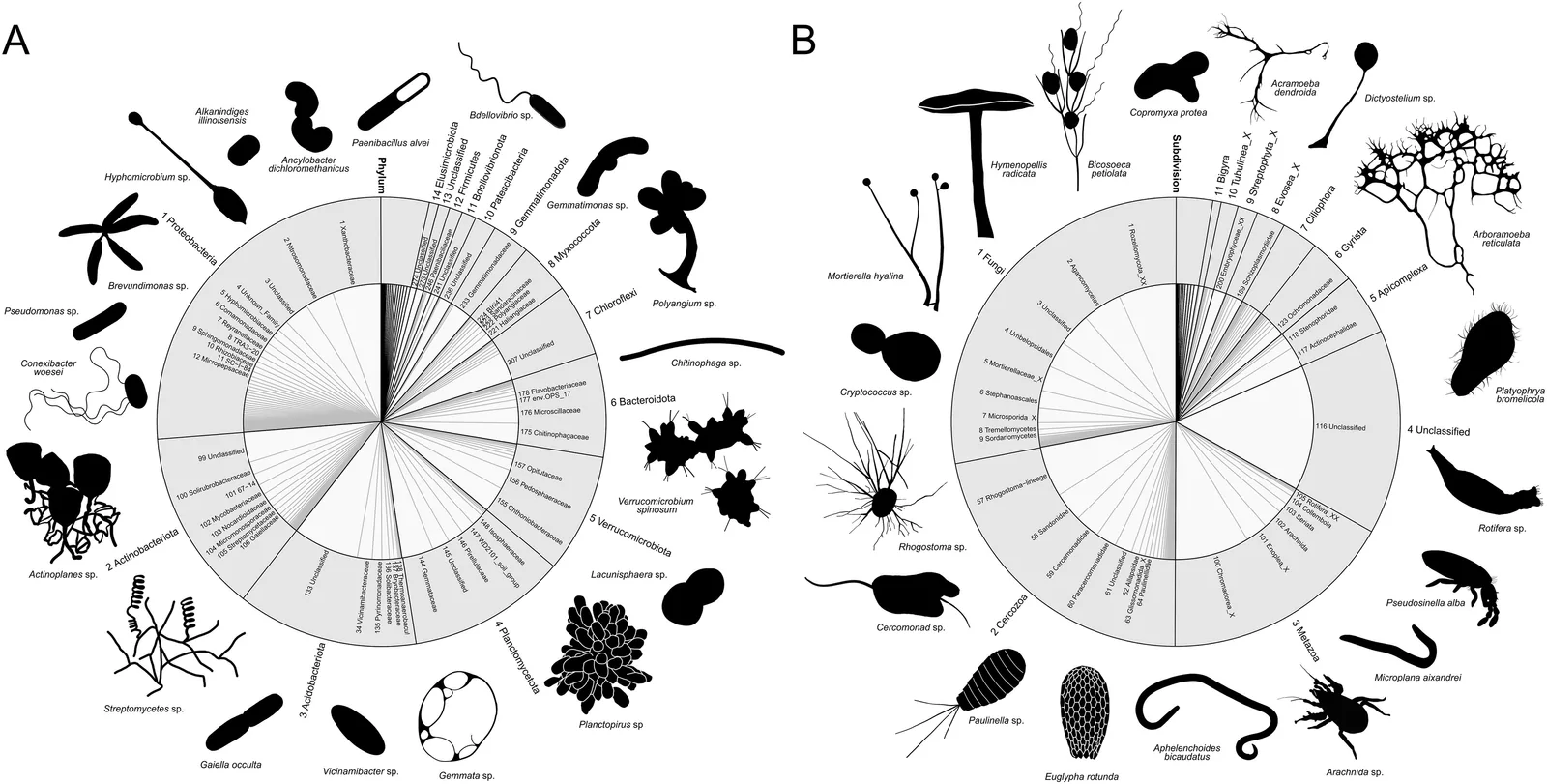
Abundance and diversity of microbial taxa within a single gram of soil. (A) Prokaryotic diversity within a single gram of soil as determined through amplicon sequencing (16S rRNA genes V4, mapping ASVs against the SILVA v138.1 database using JAMS; [289]). The inner shell of the pie diagram displays abundances of taxonomic families and outer shell abundances of phyla. (B) Eukaryotic diversity within a single gram of soil as determined through amplicon sequencing (18S rRNA genes V4, mapping ASVs against the PR2 v5.0.0 database using JAMS). The inner shell of the pie diagram displays abundances of taxonomic families and outer shell abundances of subdivisions. ASVs that could not be assigned to a subdivision are included as unclassified (even when a supergroup could be identified). Illustrations around the pie diagram showcase some of the phenotypic diversity that is observed within the soil community.

The taxonomic diversity is associated with an astonishing phenotypic diversity. In both prokaryotes and eukaryotes, species widely differ in size and shape [3] (Fig. 1). Since only few species have been imaged thus far, the known phenotypic diversity likely forms a glimpse of the actual diversity. When dissecting this phenotypic diversity, we find distinct ecological roles. Prokaryotes mostly consist of decomposers with species living from degrading organic matter [13]. This includes fast-growing species from, for example, the *Proteobacteria*, *Bacteroidota*, and *Firmicutes*, as well as many slow-growing species from, for example, the *Actinobacteriota*, *Acidobacteriota*, and *Planctomycetota*. Although less numerous, we also find parasitic, pathogenic, and predatory bacteria, with predatory *Myxococcota* and *Bdellovibrionota* being particularly abundant. In the eukaryotes, *Fungi* are the primary decomposers. In addition, we also find a high abundance of predators, including numerous microscopic protists (e.g. *Cercozoa*, *Ciliophora*, *Evosea*, *Tubulinea*) as well as mesoscopic metazoan predators (e.g. nematodes, tardigrades, rotifers, mites, springtails, and planarians) [3, 39, 40]. As predators are higher up in the food chain, they are less abundant than decomposers [41], but they nonetheless play an important role in shaping the community composition and driving community turnover [39, 42].

Mirroring the community diversity, there is also a wide diversity of abiotic conditions in soils (Fig. 2). Soils are strongly structured environments [43], consisting of many particles and pores, differing in size and shape, and trapping microbes in minute volumes of air, water, and organic debris. This strong spatial structure is also apparent in our soil sample, where conglomerates of particles and debris form a complex three-dimensional space (Fig. 2A and B). The specific surface area of a single gram of soil ranges from 10^−1^ m^2^/g in sandy soils to 10^2^ m^2^/g in clay, which means that despite the high microbial abundance only a small fraction of the surface is expected to be colonized at any moment in time [44–46]. Microbial interactions are therefore expected to be scattered in space [12]. The spatial structure also affects the distribution of resources: even at the smallest spatial scales, spanning a few microns only, there can be steep gradients in, for example, oxygen and nutrient availability [43]. Within the same soil environment, microbes therefore experience strongly different conditions [12, 47]. To accommodate, microbes move around in different ways, from flagella- and cilia-driven swimming strategies to hyphal growth and diverse forms of surface-associated locomotion [48, 49].

**Figure 2:**
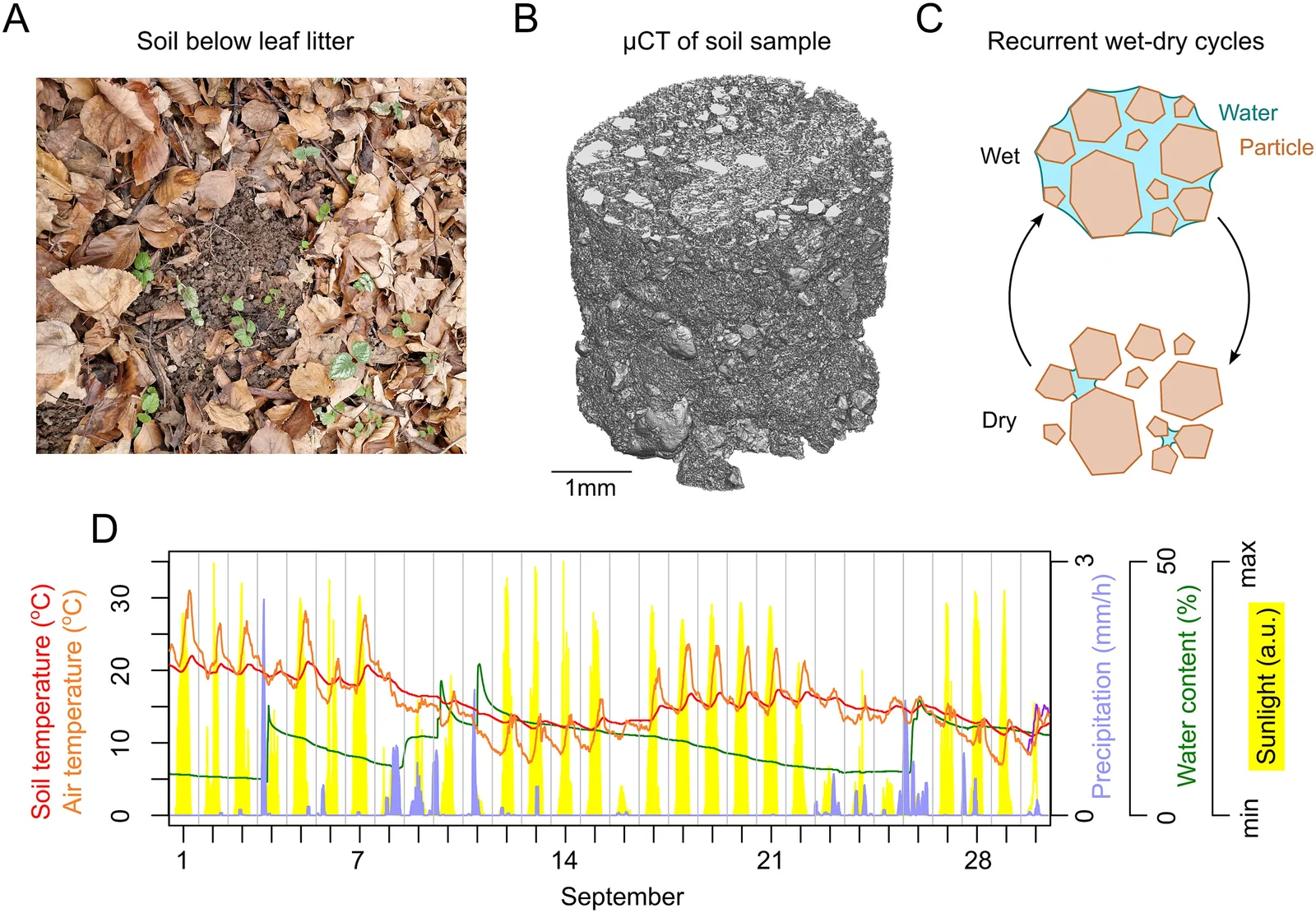
Spatiotemporal complexity of the soil environment. (A) Sample site below leaf litter at which the topsoil sample was collected for amplicon sequencing in Fig. 1. (B) μCT scan of soil sample showing the porous environment in which soil microbes reside. (C) Schematic of recurrent wet–dry cycles within the soil environment, with particles and water- and air-filled pores. During dehydration, water from large pores is expected to evaporate first, trapping microbes in small water-filled pores. (D) Daily changes in temperature (°C), precipitation (mm/h), water content (%), and sunlight (lux, normalized between lowest and highest recorded measurements) at soil sample site, underscoring the dynamic environment to which soil microbes are exposed.

Microbes are also exposed to strongly different conditions in time. At the larger spatial scale, community dynamics are entrained by seasonal changes, like the yearly influx of leaf litter stimulating decomposition. We also find recurrent changes across days in temperature, precipitation, and sunlight (Fig. 2C and D). From those, rainfall has a particularly strong impact by causing recurrent wet–dry cycles (Fig. 2C), with brief periods of near water saturation being followed by longer periods of dehydration [46]. Wet–dry cycles can drive community dynamics, with bacterial activity peaking shortly after rainfall [16, 50] and bacterivorous consumers following soon after [51–54]. On the small spatial scale, temporal changes are often driven by microbes themselves. As microbes grow, they deplete resources and accumulate waste products, slowly deteriorating their local environment [55]. Protistan and metazoan consumers, for instance, expel undigested matter that can accumulate into a local necromass [56]. The space of possible small-scale temporal interactions is almost unfathomable, but is expected to play a major role in driving community dynamics.

To **persist** (see Glossary) in a soil environment, microbes must have spatiotemporal growth benefits that compensate for any biomass losses due to both biotic (e.g. competition, predation, infection) and abiotic stressors (e.g. dehydration, osmotic shock, resource depletion) [56]. Growth benefits can for instance be associated with available resources (e.g. complex sugars, oxygen, nitrogen), interaction partners (e.g. prey, hosts), and environmental conditions (e.g. temperature, water tension, surface interfaces). Since growth opportunities come and go, microbes must persist or disperse across emergent growth opportunities in a soil environment [12]. The need to disperse is particularly acute for soils, where microbes often find themselves trapped in small porous spaces, where they effectively compete with their own progeny for resources [25, 28, 57]. An important challenge thus lies in understanding how growth opportunities emerge in time and space: as communities assemble and organize themselves, they will affect the biotic and abiotic conditions and directly or indirectly shape future growth opportunities [58–60]. Only microbes that can effectively exploit recurrent growth opportunities within these self-organizing communities are expected to persist in time [61]. How microbes do this strongly depends on their life cycle, to which I will turn next.

## Microbial life cycles

On a conceptual level, any **life cycle** (see Glossary) consists of two processes (Fig. 3A): **growth** and **propagation** [62, 63]. Growth results in the accumulation of biomass, while propagation results in its fragmentation. For growth, microbes must convert organic or inorganic matter into biomass, while for propagation this biomass needs to be partitioned into **propagules** that have the potential to grow again. **Reproduction** occurs to the extent that phenotypes before propagation can be reproduced by propagules afterwards [64–67]. The ability to reproduce a phenotype will depend on both the molecular and cellular processes that give rise to a phenotype as well as the environmental conditions that serve as a scaffold (Box 1) [68–73]. For most soil microbes, if not all, life cycle changes are intimately linked to their environment (Box 1). Bacterial biofilms might, for example, need an available substrate for growth, like particulate organic matter, while many eukaryotes depend on an air–liquid interface for fruiting body development. Life cycles should therefore ideally be monitored in their environmental context. Only phenotypes that reproducibly emerge across **generations** (i.e. heritable phenotypes) can evolve through natural selection and, by extension, we can only speak of a life cycle when there are reproducible **life stages** (i.e. **life cycle closure**) [65, 74, 75] (fn. 2, Supplementary Text 1).

**Figure 3:**
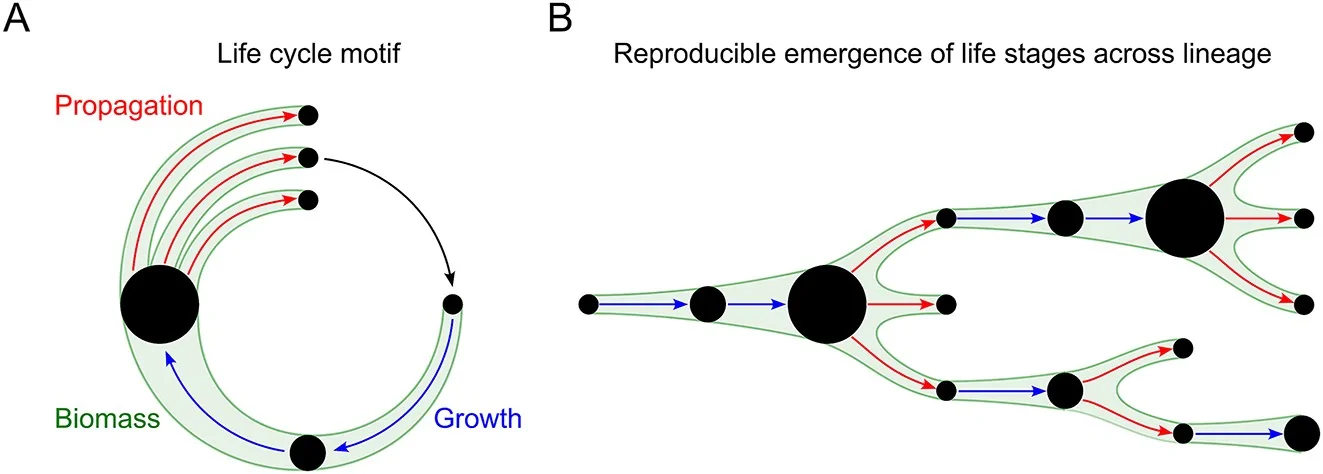
Conceptual building blocks underlying life cycle biology. (A) Conceptual diagram of a life cycle with growth (blue arrows), propagation (red arrows), life stages (black circles), and biomass changes (green). During growth, biomass increases in size. During propagation, biomass is partitioned among propagules, each of which has the potential to grow again. (B) The reproducible emergence of phenotypes along a microbe’s cell lineage makes it possible to individuate life stages and life stage transitions. Black circles are abstract depictions of a microbe’s reproducible life stages (see Fig. 4).

Microbial life cycles can be identified by following cell lineages in time and space and delineating life stages that reproducibly emerge, as apparent from their phenotypic organization (see fn. 3 in Supplementary Text 1 and Fig. 3B) [64, 65, 73, 76–78]. Such reproducible life stages either directly or indirectly benefit the processes of growth and/or propagation. At minimum, a microbe’s life cycle equals the cell cycle, where microbial growth and propagation directly follow from cell growth and division. The cell cycle shows striking differences between species [79]: some species divide through budding, others undergo symmetrical cell division, and yet others show coenocytic growth with cellularization. Despite this diversity among microbes, each of these division processes usually proceeds through a highly reproducible cascade of phenotypic changes over time.

In many soil microbes, we also find emergent life stages that go beyond the cell cycle [80]. These life stages rely on **developmental processes** that support expression of alternative phenotypic states (Box 1, Fig. 4). Some microbes, for example, express a distinctive motile state, such as the flagellated swarm cells in the *Myxogastria* [81, 82]. Swarm cells promote dispersal under moist conditions, but cannot divide [83, 84]. By the same token, microbes can also differentiate into a dormant state, like cysts and spores [85]. Soils are filled with dormant cells, which are expressed in response to adverse conditions, like starvation and dehydration. Dormant cells cannot divide, have a reduced intracellular water content, and are surrounded by a protective envelope that shields them from environmental stressors [86]. In this way, they can withstand long periods of adversity, while being able to germinate whenever conditions improve [87].

**Figure 4:**
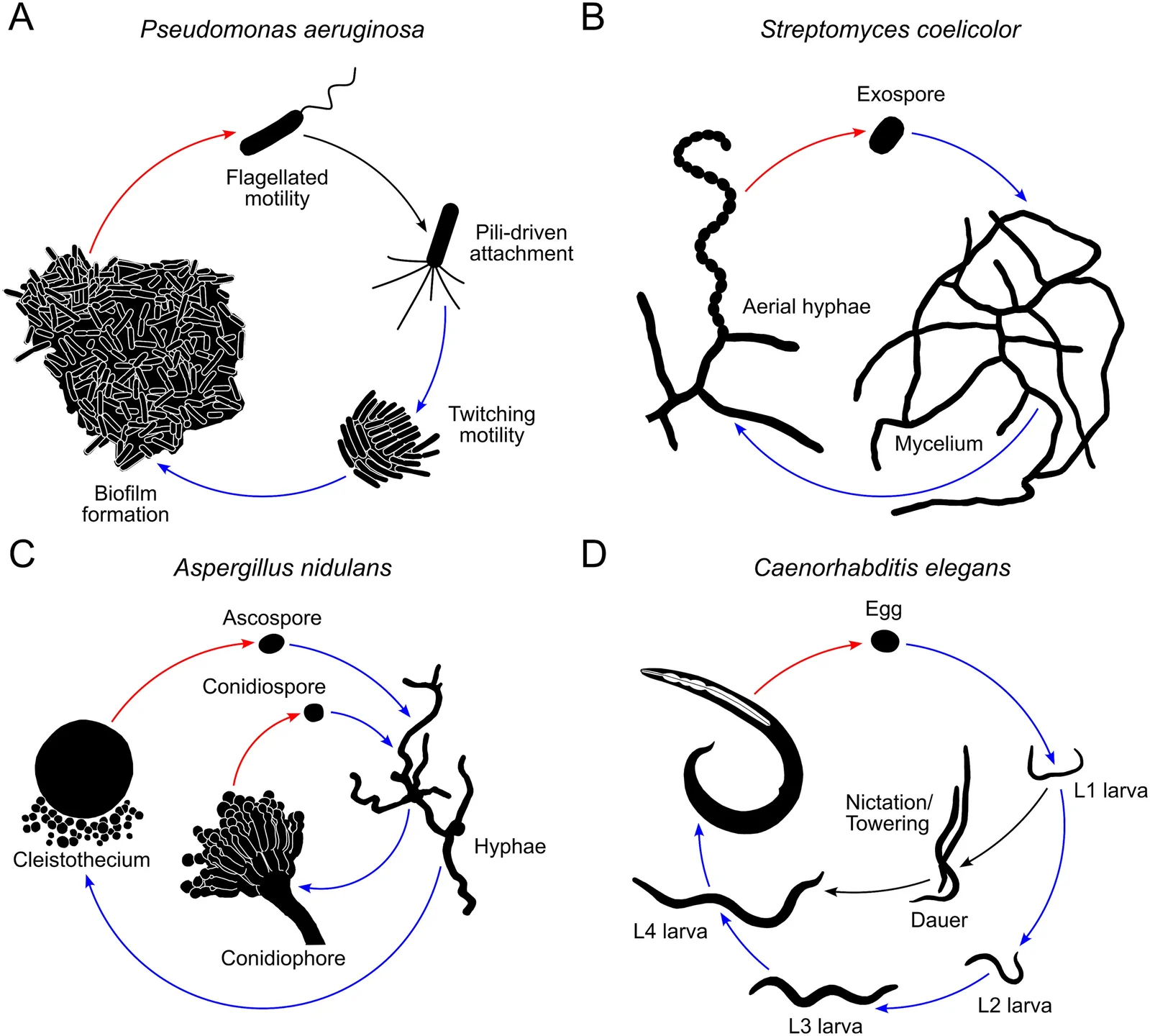
Disparate life cycle motifs in the soil. Life cycle motifs of (A) *Pseudomonas aeruginosa*, (B) *Streptomyces coelicolor*, (C) *Aspergillus nidulans*, and (D) *Caenorhabditis elegans*. Model species represent larger microbial taxa in the soil that express comparable life cycle motifs (see Box 1). Blue and red arrows indicate growth and propagation, respectively.

Emergent life stages can also consist of multiple cells (Box 1, Fig. 4) [88]. Multicellular life stages usually arise through growth, where microbial cells stay together after division, thereby accumulating biomass in the form of colonies, biofilms, mycelia, or other types of collectives [78]. These collectives often take a stereotypic appearance by the way cells grow, their spatial configuration, and the biomolecules they deposit (e.g. adhesive polysaccharides, proteinaceous fibers, eDNA) [89–91]. By forming collectives, microbes effectively trade propagation for growth: instead of partitioning after division, cells stay together and grow into a collective. As life cycles are recursive, deferring the opportunity to propagate now must benefit propagation later [92]. Many collectives therefore come with emergent benefits. Collectives can for instance have an increased resistance against abiotic and biotic stressors. *Pseudomonas aeruginosa* biofilms, for example, provide resistance to dehydration through alginate production [93] (Box 1, Fig. 4A), while *Bacillus subtilis* biofilms can withstand protistan predation [94].

Multicellular life stages can also promote growth. By forming groups that swarm, slide, or glide, bacterial collectives can, for example, grow over surfaces where individual cells cannot [95, 96]. Surface growth often depends on the collective production of compounds, like surfactants, which are expressed in a density-dependent manner through quorum-sensing signaling [97, 98]. In addition, bacteria can also secrete antimicrobials, which do not directly improve surface growth but help deter competitors [99–102]. In many cases, collectives can also promote growth by breaking down complex sugars through the secretion of costly polysaccharide-degrading enzymes (e.g. cellulases, ligninases, amylases, chitinases) [13, 103, 104]. Collective digestion is seen in many bacterial and fungal decomposers as well as several predators. *Myxococcus xanthus*, for example, kills and digests its prey by secreting a pool of digestive enzymes [105–107]. Collective predation can also take on a clear behavioral component. The amoebal predator *Cryptodifflugia operculata*, for example, collectively attacks and consumes nematodes that are many times larger in size [108].

Finally, multicellular life stages can promote dispersal. Many microbial species express fruiting bodies that rise above the surface, allowing cells to escape surface tension and disperse. Fungi in the *Ascomycota* and *Basidiomycota*, for example, produce macroscopic fruiting bodies as part of their sexual cycle, promoting spore dispersal [109]. Dispersal organs are also produced as part of the asexual cycle. *Aspergillus nidulans*, for instance, produces spore-bearing aerial conidiophores [110]. Conidiation is induced by several environmental cues, like air exposure, starvation, and light, and involves a highly reproducible process of phenotypic changes that results in a mature conidiophore (Box 1, Fig. 4C). Similar microscopic dispersal organs are found in other soil microbes as well, including, for example, aerial hyphae with exospores in the *Actinobacteriota* (Box 1, Fig. 4B) [102, 111], fruiting bodies with myxospores in the *Myxococcota* (Box 1) [112], and sporocarps in the *Amoebozoa* [113]. Despite the abundance of microscopic dispersal organs in soil environments, their effective benefits remain largely unquantified [114].

Although less common, multicellular life stages can also emerge through aggregation instead of growth. Some of the protistan predators in our soil sample, like *Dictyostelia* and *Copromyxa*, for instance produce fruiting bodies through aggregation [115, 116] (Box 1, Fig. 5A). Upon starvation, amoebal cells coordinate their movement toward a focal point in the environment, where they aggregate and develop into a fruiting body. Aggregative life cycles evolved at least seven independent times among soil protists [117] and combine the best of two worlds: their single-cell state allows protists to consume bacteria in even the smallest spatial pores, while their multicellular state supports dispersal over long distances. Even *Metazoa* can express aggregative life stages: in *Caenorhabditis elegans*, for instance, worms can aggregate to form towers that protrude from the surface and promote dispersal [118] (Box 1, Fig. 4D). Aggregation underscores the tension between the small spatial scale at which microbes interact within the soil and the large spatial scale at which they need to disperse. The aggregative life cycle also underscores the importance of lineage tracing: only by following microbes over time does the importance of aggregation for life cycle closure become evident.

**Figure 5:**
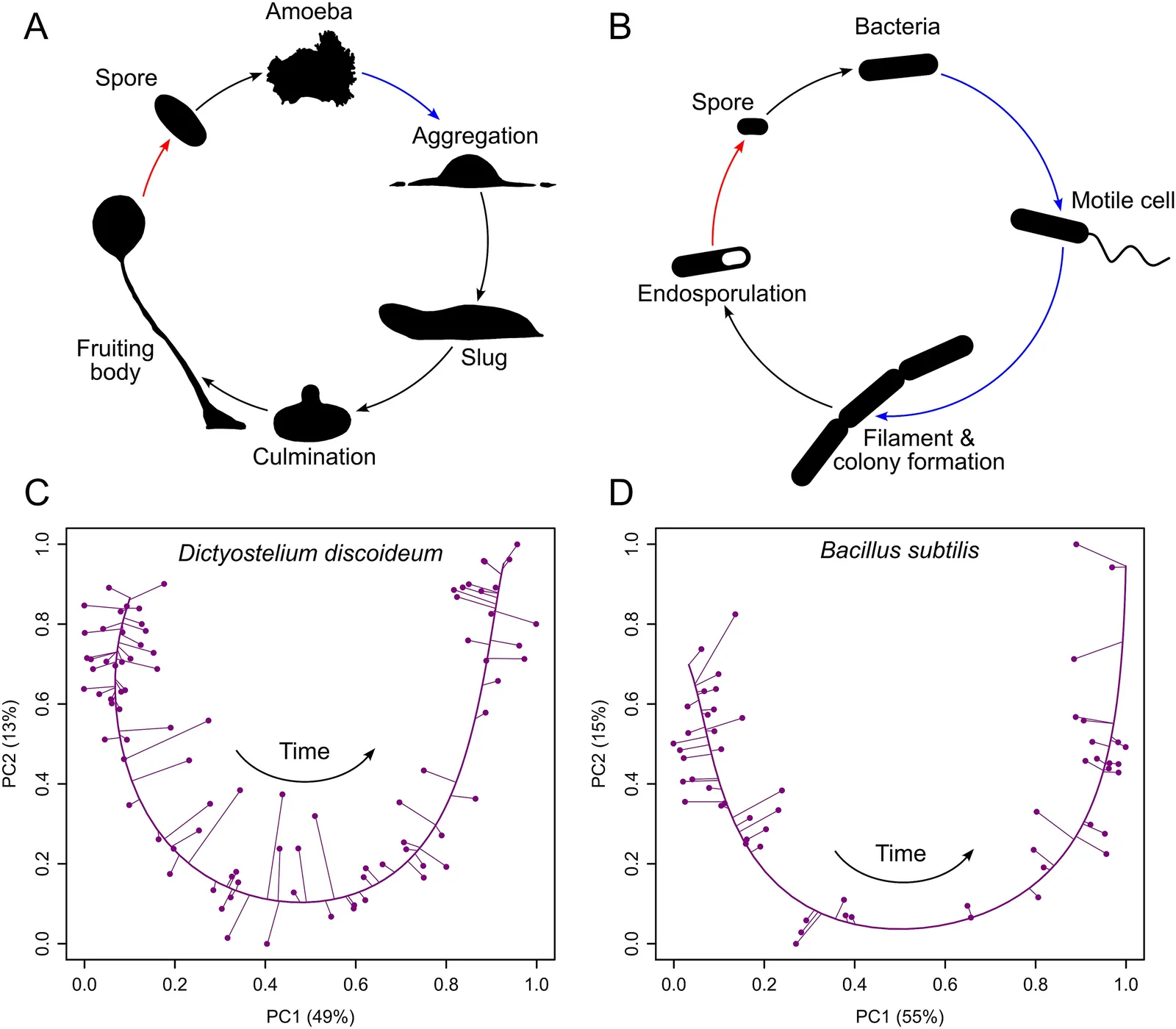
Life cycle trajectories in *Dictyostelium discoideum* and *Bacillus subtilis*. (A, B) Life cycle motifs of *D. discoideum* and *B. subtilis*, respectively, with both growth (blue arrows) and propagation (red arrows) stages. (C, D) Life cycle trajectories represented by temporal expression data in *D. discoideum* and *B. subtilis*, respectively. Dots represent individual gene expression profiles. Temporal expression data were obtained from Katoh-Kurasawa and colleagues [121] for *D. discoideum* and Nicolas and colleagues [290] for *B. subtilis*. Life cycle trajectories are derived from expression data using an autoencoder approach as described by van Gestel and colleagues [119].

By expressing distinct life stages, whether they consist of single or multiple cells, microbes effectively partition phenotypes related to growth and propagation across their life cycle (see fn. 4 in Supplementary Text 1). These phenotypes can be expressed in a coordinated and reproducible manner, giving rise to stereotypical life stages and life stage transitions. Stereotypical life stages are often associated with broader taxonomic groups (Box 1), like endospores typifying the *Firmicutes* and aggregative fruiting bodies the *Dictyostelia*. In some species, the partitioning between growth and propagation is well quantified. In *B. subtilis*, for example, we find clearly distinguishable life stages from growth to dormancy [119]: Early in the life cycle, *B. subtilis* cells express a solitary motile life stage, then—following resource depletion—they can form slow-growing adhesive colonies and finally develop into endospores (Box 1, Fig. 5B). Similarly, during the aggregative life cycle of *Dictyostelium discoideum*, we find stereotypical changes from amoebal growth to fruiting body development, where cells aggregate, form slugs, culminate, and sporulate [120] (Box 1, Fig. 5A). These life cycle changes are associated with highly reproducible gene expression changes in time (Fig. 5C and D) [119, 121, 122]. Similar stereotypical life cycle trajectories are expected to exist for most soil microbes, but often remain unmapped (fn. 5, Supplementary Text 1) (Box 1).

The presence of reproducible life stages does not imply that life cycle changes are deterministic or irreversible. Depending on the environmental conditions, **individual** microbes can express many different instantiations of the same life cycle motif, where the timing and transition between life stages differ. Even when exposed to the exact same environmental conditions, cells might enter different life stages [123]. Not all *B. subtilis* cells become adhesive upon resource depletion or enter endosporulation when starved (Box 1, Fig. 5B) [124–126]. Similarly, in response to starvation, some *Dictyostelia* species might not only develop fruiting bodies (Box 1, Fig. 5A), but also produce microcysts or engage in syngamy leading to macrocyst development [127, 128]. The probability by which cells enter these different life stages depends on the environmental conditions [129, 130]. Stochastic life cycle transitions go hand in hand with phenotypic heterogeneity within microbial **populations**. Such heterogeneity can be adaptive when microbes are exposed to unpredictable and antagonistic selection pressures in their environment (fn. 6, Supplementary Text 1) [94, 131], but it could also simply result from a limited developmental control. In the end, deterministic life cycle changes—as we know them from metazoans (e.g. *C. elegans* in Box 1)—rely on developmental processes that precisely control and canalize phenotypic changes over time, such that phenotypes emerge in the same order over and over again. For many microbes such determinism might be counterproductive, but for others it might simply be unattainable (fn. 7, Supplementary Text 1).

In sum, despite living in the same environment, soil microbes express widely different life cycle motifs. These **disparate life cycles** are linked to diverse taxonomic groups, which often express unique life stages that typify their growth and propagation. Life cycles unfold over widely different spatial and temporal scales: slow-growing *Fungi* with meter-long hyphal networks might fruit only once a year, while their mycelium may persist for decades; micron-sized *Proteobacteria* might divide several times a day, while sporulating bacteria might only become active several times a year [132]. Each life cycle motif provides an effective way of getting by in soils and is the product of both the **environmental scaffold** needed for life cycle closure and the **developmental processes** underlying life cycle progression (Box 1). Environmental conditions are important for both supporting the expression of individual life stages as well as life stage transitions, with many developmental processes being induced in response to environmental cues (e.g. resource conditions, temperature, dehydration, light). Changes in either the environment or development, like genetic changes (fn. 8, Supplementary Text 1), could alter life cycle dynamics and thus the ability of microbes to persist within a soil environment. To understand why some microbes manage to persist in soils and others do not, we have to study how their life cycles emerge within the soil environment and examine how, when, and where microbes grow and propagate.

## Coupling of microbial life cycles

To understand how life cycles emerge, we must also consider how microbes interact with each other, as these interactions can strongly shape life cycle dynamics. That is, life cycles do not emerge in isolation, but are coupled, such that the growth and/or propagation dynamics of one microbe are connected to those of others (fn. 9, Supplementary Text 1) (**life cycle coupling**). This dynamical coupling arises from ecological interactions within the soil [133–136] and often occurs in a life stage-dependent manner, with life stages affecting how, when, and where microbes interact. Most interactions are thought to result in a form of growth coupling, where the growth of one microbe either directly or indirectly modifies that of others. Growth coupling may, for example, occur through compounds that are released into the environment. Auxotrophic bacteria can, for instance, exploit amino acids produced by prototrophic bacteria for growth, thereby avoiding the costs of amino acid biosynthesis [137–139]. Similarly, also cascading redox reactions can lead to growth coupling (Fig. 6A) [140]: nitrite-oxidizing bacteria, such as *Nitrobacter*, can for example depend on nitrite produced by ammonia-oxidizing bacteria, such as *Nitrosomonas*, which oxidize ammonia to nitrite [141]. In many cases, compounds are released during specific life stages. During collective growth, for instance, microbes often produce complex sugars to mediate cell–cell adhesion (Fig. 6B): *Pseudomonas* biofilms often produce alginate and cellulose [142], *Bacillus* biofilms produce levan and poly-γ-glutamate [143], and *Aspergillus* mycelia produce galactomannans [144]. These complex sugars accumulate in the local environment and serve as a food source for degraders, thereby coupling the local growth of producers and degraders [145].

**Figure 6:**
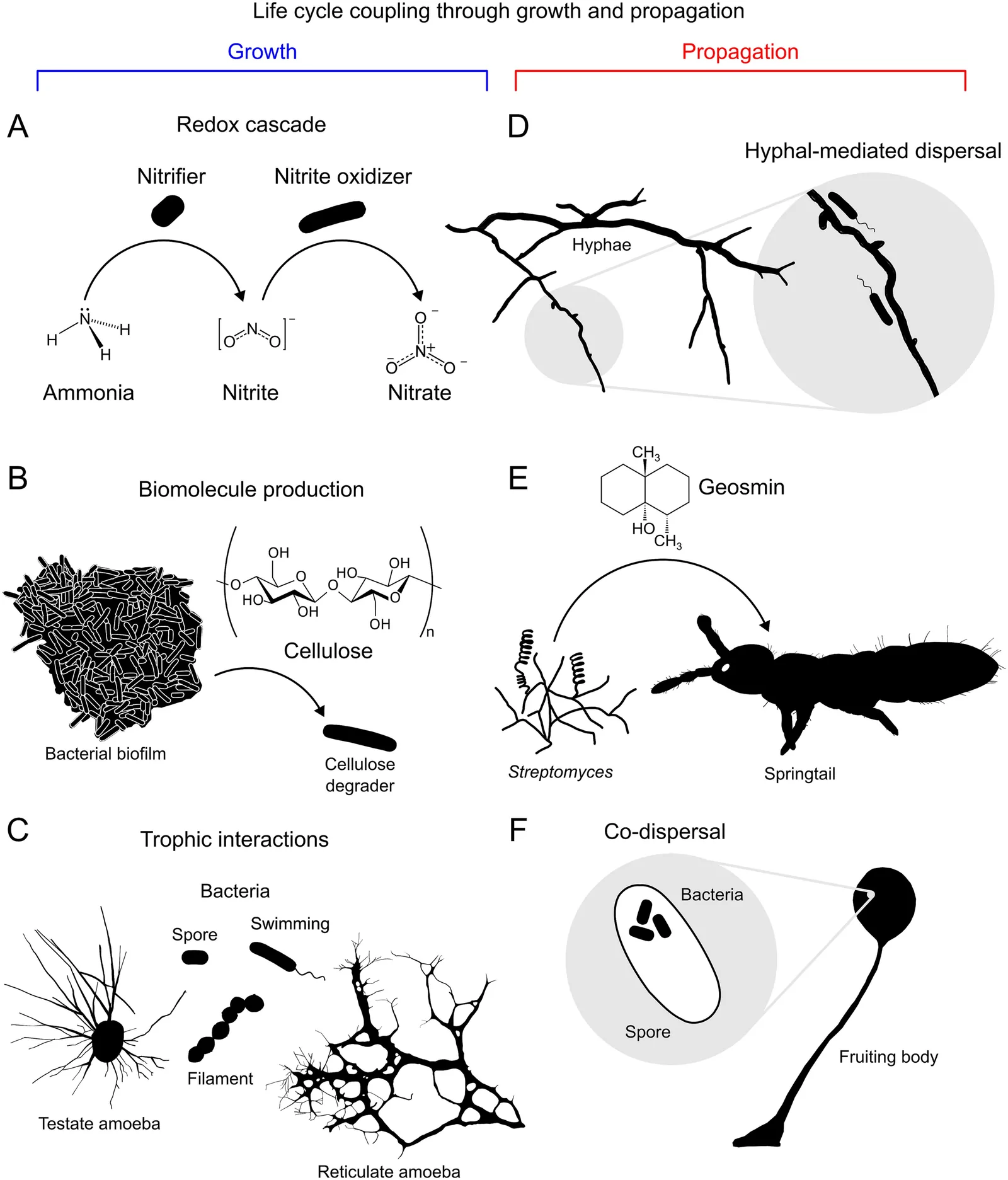
Life cycle coupling through life stages affecting growth and propagation. Life cycles can be coupled in versatile ways affecting growth and/or propagation. Growth can e.g. be affected by (A) redox cascades, (B) complex sugar production, and (C) trophic interactions. Propagation can e.g. be affected by (D) passive dispersal interactions, (E) dispersal organs, and (F) co-dispersal events.

The growth of one species may also directly be coupled to that of others through trophic interactions, like predator–prey and host–parasite interactions (Fig. 6C). In these interactions, one species consumes or infects the other. In our soil sample, we find many such interactions: focusing on predation, there are both predatory bacteria (*Myxococcota* and *Bdellovibrionota*) and protists (e.g. *Cercozoa*, *Ciliophora*, *Evosea*), as well as many mesoscopic consumers (e.g. nematodes, mites, springtails, tardigrades) [146]. Fueled by metabolic dependencies, the growth of these predators is effectively locked to that of their prey [147, 148]. Many predator–prey interactions occur in a life stage-dependent manner. Some prey-specific life stages can, for example, resist predation: collective life stages can be too big for ingestion, like *Bacillus* and *Pseudomonas* biofilms outsizing their phagocytic predators [94, 149], while dormant life stages, like cysts and spores, could resist digestion [150, 151]. Life stages might even reverse the interaction between predator and prey, by enabling prey species to kill their predator (e.g. pathogenic life stages) [152, 153]. Predator-specific life stages, in turn, often affect when and where predator–prey interactions occur. Bacterivorous protists can, for example, switch between flagellated and amoeboid life stages, enabling them to forage in both water-filled pores as well as along surfaces [7]. Other protists, like *Myxogastria*, can transition from small amoeboid to larger plasmodial life stages, likely affecting where they occur and what prey items can be engulfed [154].

Besides growth, life cycles can also be coupled through propagation, where one species affects the propagation of another (e.g. phoresy). In many cases, propagation is affected in a passive manner, without active signaling. Fungal hyphae can, for example, promote bacterial dispersal by providing a water-coated scaffold along which bacteria can swim in unsaturated soils (Fig. 6D) [155, 156]. In other cases, microbes can actively mediate ecological interactions that promote dispersal by inducing specific life stages. Fungal fruiting bodies can, for instance, promote vector-mediated spore dispersal by emitting volatile compounds that attract insects [157, 158]. Fungal spores might also express appendages that improve attachment and thus dissemination over long distances [159]. Similarly, during sporulation, *Streptomyces* secrete a terpenoid compound called geosmin [160], responsible for the earthy smell of soil. Geosmin attracts springtails—a common group of soil invertebrates (*Collembola*, Fig. 1)—which promote the dispersal of *Streptomyces* exospores by touching their aerial hyphae (Fig. 6E) [161]. By improving dispersal, fruiting bodies also affect colonization dynamics, differentiating early from late colonizers and shaping which microbes encounter each other during colonization [162, 163].

When microbes grow on top of or inside each other, dispersal of one microbe might directly be coupled to that of the other: nematodes could for instance promote the dispersal of their symbiotic gut microbes [164, 165], while protists might carry endosymbionts that co-disperse [166]. In the case of *D. discoideum*, several endosymbionts were shown to survive inside spores, allowing them to co-disperse through fruiting body development (Fig. 6F) [166–169]. Some species might even show coupling of both growth and propagation, where they not only co-disperse but also grow together, fully aligning their reproductive fates. For example, the chlamydial species *Reclusachlamydia socialis* resides intracellularly throughout the life cycle of *Dictyostelium giganteum* and appears to lack an extracellular infectious stage, coupling its fate to that of the host [170]. Such coupling can ultimately lead to the emergence of obligate endosymbiotic relationships [171]. Indeed, several protists—including both amoebae and ciliates—host bacterial endosymbionts with fully synchronized life cycles [172, 173]. How common obligate endosymbionts are in soils remains largely unknown, but given the large number of species that have never been isolated or imaged before, they might be more widespread than expected.

The above examples of life cycle coupling highlight the importance of life cycle biology for community ecology. Because microbial interactions often occur in a life stage–specific manner, changes in life cycle dynamics are expected to affect species dynamics as well. This makes it difficult to decouple life cycle biology from community ecology: environmental conditions that affect community dynamics might do so by modulating the abundance of different life stages, which affect what species interactions are realized within a soil environment. Community dynamics might therefore not only reflect which species interact, but also which life stages interact, which has major implications for both the dynamics that could emerge and the way we interpret them [174, 175]. Life cycle biology not only affects species interactions, but species interactions also affect life cycle biology: many life stages are supported by specific interactions, which contribute to the environmental scaffold needed for life cycle closure. This support is immediately evident for some species, like predators that rely on their prey for growth, but likely plays an important role for many other species as well, as highlighted above [161, 176, 177]. The species interactions that matter for life cycle closure may furthermore change with life stage. *Dictyostelia* species depend on bacterial prey for growth, but may rely on meso- or macrofauna for spore dispersal [178–180]. To understand how ecological interactions contribute to the persistence of microbes within a soil environment, we should therefore study what interactions occur along their life cycles and examine whether these interactions either hinder or support life cycle progression.

## Soil communities: a constellation of coupled life cycles

Starting from a gram of soil collected on our campus, I reflected above on the diverse life cycles within the soil. Soil communities could be viewed as a constellation of disparate life cycles that are coupled in time and space (Fig. 7). This constellation is a back-and-forth between development and ecology, where developmental processes bring about disparate life cycles, supported by an environmental scaffold, while ecological processes mediate their coupling [181, 182]. The disparate life cycles are the product of millions of years of evolution within each of the broader taxonomic groups and are widely distinct in kind—having unique life stages and life stage transitions. The biomass within the soil is partitioned among those broader taxonomic groups, each group persisting through its ability to compensate for biomass losses through growth, where life cycles mediate the flow of matter across life stages and generations (Fig. 7). Although I covered only a few life cycle motifs here (Box 1, Figs 4 and 5), with this essay I aimed to underscore the central importance of life cycle biology for understanding how microbes persist and interact within soils. The link between life cycle biology and soil ecology raises many new research questions, related to life cycle ecology (Box 2), and provides exciting opportunities for future research in soil microbiology. I will reflect on some of these opportunities in the concluding paragraphs below.

**Figure 7:**
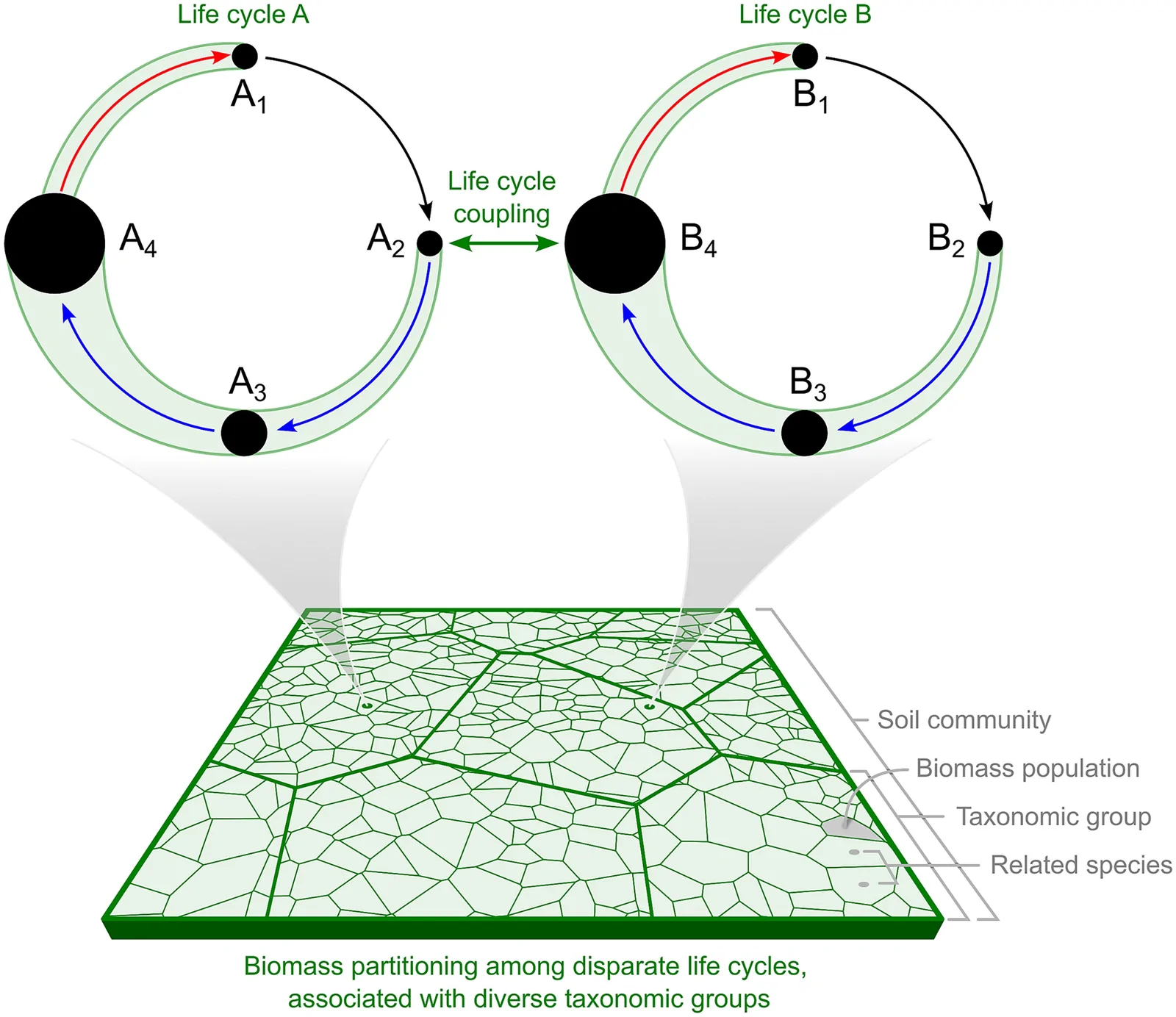
Conceptualization of soil communities as a constellation of coupled life cycles. Soil communities consist of broader taxonomic groups that express disparate life cycle motifs (e.g. Figs 4 and 5). These groups consist of many different species, each expressing variants of the same life cycle motif. Life cycles determine how biomass is distributed across life stages and generations. Life cycle coupling affects how biomass is distributed between taxonomic groups, as represented by the two-dimensional Voronoi landscape (Fig. 6).

Life cycle biology could guide the development of experimental assays. Since life cycles emerge from the interaction between microbes and their environment, experimental assays run the risk of imposing life cycles artificially by directing how microbes grow and propagate: e.g. bacterial soil communities are often propagated through serial passaging, which supports growth but not dormancy; for microbes that express fruiting bodies, assays rarely include a dispersal stage, masking the impact of dispersal; and, for microbes that express sessile and motile life stages, assays often favor one over the other. Instead of adapting experimental assays to the life cycle biology of microbes, most assays thus impose a particular life cycle regime and examine how microbes adapt to this regime [119]. This approach makes it difficult to determine what environmental conditions matter for life cycle closure in soils, including the relevant spatial and temporal scales at which we should study them. The link between life cycle biology and soil ecology argues for an opposite approach that starts from the life cycle biology of a microbe and builds a soil-like environment that supports spontaneous life cycle closure, while preserving the ability to monitor microbes as they grow and propagate. For a life cycle to persist, this environment should not only support the emergence of individual life stages but also their benefits, favoring their expression with ongoing propagation [183–185]. Recent advances in building synthetic soils provide a promising way forward in developing such experimental assays [22].

The discontinuous soil environment—with solid, water, and air phases—might play a particularly important role in supporting disparate life cycles. On the small spatial scale, wet–dry cycles could, for example, mediate phenotypic transitions between sessile and motile life stages in bacteria: under dry conditions, bacteria might be locked in space, inducing biofilm formation, while under wet conditions, they could swim and disperse through flagellated motility [27, 93, 186, 187]. Wet–dry cycles also affect the presence of air–liquid interfaces, which can support fruiting body development. Similarly, a discontinuous distribution of resources, like particulate organic matter, could drive transitions in and out of dormancy and support a need for dispersal, with growth opportunities emerging sporadically [12]. To determine what soil properties matter for life cycle closure across microbial taxa, we need systematic assays where microbes are exposed to many soil-like environments that differ in a quantitative manner, while observing how these conditions affect the expression of individual life stages as well as life stage transitions. Ideally, such assays should be performed at scale, making it possible to modulate several properties in parallel and in a combinatorial fashion. These assays would form a much needed ecological counterpart of genetic assays that focus on the molecular and cellular processes underlying life cycle biology [188–190].

In many cases, if not most, spontaneous life cycle closure cannot be attained by microbes in isolation, but instead relies on the presence of other community members [191–193]. In the laboratory, these dependencies can be masked by growing microbes under axenic growth conditions, in which essential resources are supplied and waste products are removed experimentally. Axenic growth conditions are ideal for studying the molecular and cellular processes underlying life cycle biology, but make it difficult to study how life cycles emerge in soils. Axenic growth conditions also mask functional benefits that are associated with certain life stages, like the benefits of bacterial biofilms in resisting protistan predation. Microbial life cycles should therefore ideally be studied in the context of soil communities, where interactions between species can support the emergence and benefits of individual life stages. Since species interactions can span widely different taxonomic groups, this requires experimental assays that accommodate the taxonomic breadth of soil communities [194] (Fig. 1). To develop such assays, we should bridge existing research domains (e.g. bacteriology, mycology, protistology, and nematology) and rethink the spatiotemporal scale of our assays to support, among others, slow- and fast-growing, small and large, and mobile and sessile soil species.

Communities do not only affect life cycle dynamics, but life cycles also affect community dynamics (Fig. 7). By expressing distinct life stages, microbes can affect how, when, and where species interact. When certain life stages are absent from the community—either because environmental conditions do not support their expression or because taxa that express them cannot persist—the associated biomass will be redirected to other life stages, which is expected to affect species interactions and thus community dynamics. To study the impact of life cycle biology on community ecology, we should therefore ideally manipulate life stages and quantify their effect on species interactions. For instance, by purposely removing (or modulating) certain life stages through genetics, like those conserved across broader taxonomic groups (e.g. motile states, microcysts, endospores, biofilms, fruiting bodies), we can determine how they affect the fate of microbial taxa within soil communities and the spatiotemporal community organization. Such removal experiments would form a life-stage equivalent of species-removal experiments [195, 196], in which life stages, not species, are used as the unit of perturbation for measuring community effects. By taking a life cycle perspective and targeting broadly conserved life stages, we can resolve how disparate life cycles are coupled in time and space and what brings about persistent constellations of coupled life cycles in soil environments.

### Glossary

**Population.** A set of individuals belonging to the same taxonomic group, e.g. species, that occurs in a given environment.

**Persistence.** The ability of a population to compensate for biomass losses caused by biotic (e.g. predation, infection) or abiotic stressors (e.g. dehydration, starvation) through growth.

Box 1 Developmental processes underlying life cycle progression in model species.

The developmental processes underlying life cycle progression have been studied in detail for a small number of model species, each supported by their own research community, providing an in-depth understanding of the cellular and molecular processes that underlie phenotypic change. Although these models express widely different life cycles, their underlying development shows clear commonalities [88]. For most species, life cycle progression depends on the environment. Resource depletion for instance commonly triggers life stage transitions, like the onset of endosporulation or fruiting body development. Such life stage transitions can take considerable time: *Bacillus subtilis* needs 6 to 7 h to undergo endosporulation; *Dictyostelium discoideum* needs about 24 h to complete fruiting body development; and *Aspergillus nidulans* takes 15 h for conidiation. To ensure the timely expression of these phenotypes, induction should occur well before microbes are fully starved and is therefore tightly regulated. In many cases, induction relies on second messengers (e.g. (p)ppGpp, c-di-GMP, cAMP), which are controlled by enzymes that make them (e.g. cyclases) and break them down (e.g. phosphodiesterases) in response to environmental cues, like starvation signals [197]. These second messengers typically act in conjunction with transcriptional regulators that regulate downstream genes. Besides environmental cues, also quorum-sensing signals play an important role in inducing life stage transitions, allowing microbes to tune the timing of transitions depending on the population density [198–201]. Once induced, regulatory cascades guide the phenotypic changes that are associated with a life stage transition. Such changes result in a turnover of matter and a repurposing of biomass, oftentimes being associated with cell lysis and autophagy: in *Pseudomonas aeruginosa*, cell lysis supports the production of eDNA, which is important for biofilm integrity; in *B. subtilis*, the mother cell lyses to support endosporulation; in *Dictyostelium discoideum*, stalk cells lyse to support fruiting body development; and in *Aspergillus nidulans*, autophagy plays an important role in conidiation. In contrast to the often-detailed understanding of the regulatory cascades, how biomass is exactly remodeled often remains relatively understudied. Below, I briefly summarize the life cycle motifs expressed by seven well-studied model species.

***Pseudomonas aeruginosa***. Although *P. aeruginosa* is mostly studied as an opportunistic pathogen, it is part of the *Pseudomonadales*, a taxonomic order that is broadly found in soils. *P. aeruginosa* is thought to function as a secondary consumer, growing on compounds released by primary decomposers of plant matter. The life cycle of *P. aeruginosa* is composed of both a motile and a sessile life stage [202]. Upon surface contact, motile cells can rapidly increase c-di-GMP concentrations, leading to the activation of FimW and the assembly of type IV pili, mediating surface adhesion and twitching motility [203]. On the surface, cells can divide and only those with a high c-di-GMP concentration remain attached. Cells that remain surface associated can form a biofilm by producing extracellular polysaccharides (e.g. Psl, Pel) [204], with among others alginate (*algC*) [205], as well as rhamnolipids and eDNA [206]. These biofilm components affect the integrity of the biofilm and surface adhesion. Biofilm formation depends on several quorum-sensing systems (e.g. Las, Rhl) and a series of two-component systems that undergo sequential activation as the biofilm matures (e.g. BfiSR-BfmRS-MifRS) [207]. The *N*-butyryl-l-homoserine lactone from the Rhl system has for instance been linked to the regulation of rhamnolipid, Psl, and pyocyanin, the last of which is linked to eDNA production by causing cell lysis through autotoxicity [208]. As the biofilm grows, steep gradients emerge in both resource and oxygen availability, which drive spatial patterning, including, for example, the occurrence of cell lysis [209]. Cells within the biofilm can also disperse away, which is associated with a decrease in the c-di-GMP concentration—driving flagellated motility—and the expression of matrix-degrading factors that degrade the biofilm matrix [210, 211], effectively closing the life cycle.

**Life cycle.** Repetitive cycle of microbial growth and propagation: during growth, a microbe increases in biomass by assimilating resources; during propagation, a microbe partitions its biomass to propagate. Life cycles can be individuated by following microbial lineages in time and space and delineating the processes of growth and propagation.

**Life stage.** A stage in the life cycle associated with the coordinated expression of phenotypes that can be individuated in time and space. Stereotypic life stages are those that appear in a reproducible manner across generations.

**Growth stage.** Life stage where a microbe increases in biomass (i.e. size).

**Propagation stage.** Life stage where a microbe partitions biomass to produce one or more propagules with the potential to grow again.

**Propagule.** Phenotypic state that supports propagation, like spores, cysts, and reproductive gametes.

**Development.** Molecular and cellular processes that support the emergence of a life cycle, irrespective of whether life cycle changes are reversible or deterministic.

**Environmental scaffold.** Environmental conditions, both biotic and abiotic, that support the emergence of a life cycle, including both individual life stages and life stage transitions.

**Individual.** Single spatiotemporal instantiation of a microbe’s life cycle.

**Generations.** Consecutive spatiotemporal instantiations of a microbe’s life cycle.

**Reproduction.** Repeated emergence of phenotypes across generations, supported by developmental processes mediating life cycle changes and the environmental conditions that serve as a scaffold.

**Life cycle closure.** Ability of a microbe to complete its life cycle in a given environment with the spontaneous emergence of each individual life stage.

**Life cycle coupling.** The coupling of growth and/or propagation dynamics between microbial life cycles. Coupling can arise through a broad range of ecological interactions (e.g. predation, infection, phoresy, endosymbiosis) and often occurs in a life stage–specific manner, with life stages affecting how, when, and where microbes interact.

**Disparate life cycle.** Life cycle motif associated with a broader taxonomic group, being different in kind from those of other taxonomic groups and associated with stereotypical life stages and life stage transitions that typify the group.

**Soil community (compositional viewpoint)**. A set of populations belonging to different taxonomic groups, e.g. species, that occurs in a given environment.

**Soil community (life cycle viewpoint)**. A constellation of disparate life cycles that are coupled in time and space—through direct and indirect ecological interactions—in a way that all persist.

***Bacillus subtilis*** *. Bacillus subtilis* is part of the *Firmicutes*, a phylum of Gram-positive bacteria that are widely found in soils and known for their ability to form endospores. The life cycle of *B. subtilis* is composed of several life stages: a motile stage with flagellated cells, a sessile stage with filamentous cells producing biofilm, and a dormant stage with endospores [119]. Life cycle progression is not deterministic but follows from resource depletion. As such, *B. subtilis* life stages are typically thought of as alternative phenotypic states. The motile life stage depends on the expression of the *fla/che* operon, which encodes >30 proteins supporting flagellar assembly and chemotaxis [212]. *fla/che* expression is controlled through multiple transcription factors (e.g. CodY, SwrA, SinR) and sigma factors (σ^A^, σ^D^), making sure that cells only produce flagella when needed. CodY for instance represses *fla/che* expression when intracellular GTP concentrations are high—indicative of nutrient-rich conditions [213, 214]. When conditions worsen, they could trigger the stringent response, leading to the conversion of GTP into (p)ppGpp by RelA, lowering the GTP concentration and releasing the CodY-mediated repression of the *fla/che* operon [215]. Upon biofilm formation, other regulators in turn repress *fla/che* expression [216], effectively individuating the motile and sessile life stages. Biofilm formation is regulated through Spo0A [217], which is activated through a phosphorylation cascade that integrates both environmental cues—through histidine kinases (KinA-D)—and quorum-sensing signals—through phosphatases (Rap) [218]. When the level of phosphorylated Spo0A is sufficiently high, it releases the SinR- and AbrB-mediated repression of biofilm-related operons, including the *tapA-sipW-tasA* and *epsA-O* [219, 220]. These operons underlie the production of TasA fibers and extracellular polysaccharides that, in addition to other components (e.g. BslA, poly-γ-glutamic acid), comprise the biofilm matrix [221–223]. While producing biofilm matrix, the SlrR–SinR complex also represses flagellated motility (i.e. *fla/che* expression) and the production of autolysins (*lytA*, *lytF*), leading to filamentation [224]. When resource depletion increases the levels of phosphorylated Spo0A further, cells can enter the endosporulation process [225, 226]. During endosporulation, cells undergo asymmetric cell division creating a large mother cell and a smaller forespore. The forespore is engulfed by the mother cell, where it matures into an endospore that is released into the environment upon autolysis of the mother cell. The spore coat consists of several layers—basement layer, inner coat, outer coat, and crust—important for stress resistance [227]. Endosporulation is controlled by a cascade of sigma factors: sigma factors E and K are sequentially activated in the mother cell, while sigma factors F and G are activated in the forespore [228]. Once released, endospores can germinate whenever conditions improve, closing the *B. subtilis* life cycle.

***Streptomyces* spp.**  *Streptomyces coelicolor* and *Streptomyces venezuelae* are model species within the *Actinobacteriota*, which are abundantly found in soils and known for their multicellular life cycle [102]. During growth, cells produce a vegetative mycelium that consists of branching filamentous cells, growing at their tips, generating a hyphal network [111]. This mycelium can produce aerial hyphae that are protected by a hydrophobic sheath and protrude from the surface to undergo sporulation at their apical cells, generating spore chains [111, 229]. Spores can disperse and resume growth under beneficial conditions. The timing of aerial hyphae and spore chain formation is precisely regulated. As for other bacterial life cycles, the second messenger c-di-GMP plays an important role in regulating this process [230, 231]. Upon germination, the c-di-GMP concentration is high and subsequently declines [232]. By assembling into a tetramer, c-di-GMP supports the dimerization of the transcriptional regulator BldD, which can thereby act as a suppressor [233]. In this way, BldD blocks aerial hyphae formation during vegetative growth. Once c-di-GMP concentrations decrease below a critical concentration, the BldD-mediated repression of aerial hyphae formation is released, resulting in the expression of several sigma factors (e.g. *bldN*, *sigH*, *whiG*), regulatory genes (e.g. *bldC*, *bldM*), and effector genes (e.g. *ftsZ*, *ssgA*, *ssgB*) [234]. Besides the role of c-di-GMP in regulating BldD, it also mediates the sequestering of the sporulation sigma factor σ^WhiG^ from the RNA polymerase by the anti-sigma factor RsiG, by forming a complex with RsiG-σ^WhiG^ [235]. c-di-GMP thereby blocks sporulation when concentrations are high. As the affinity of c-di-GMP for the RsiG-σ^WhiG^ complex is higher than its affinity for BldD, the repression of sporulation is only released after that of aerial hyphae formation, ensuring their sequential induction. During sporulation, the c-di-GMP concentration again increases, resetting conditions for the next life cycle [232].

***Myxococcus xanthus*** *. Myxococcus xanthus* is part of the *Myxococcota*, an important phylum of predatory bacteria that are known for their unique life cycle [236]: during growth, myxobacteria prey on bacterial cells and when starved they aggregate into fruiting bodies that carry thousands of myxospores [237]. Both life stages rely on collective behaviors [238, 239]. During predation, cells express a form of swarming motility. In this social form of motility (i.e. S-motility), cells move over surfaces as groups using type IV pili, while secreting EPS [107, 240]. When these swarming groups invade bacterial prey colonies, they start lysing and consuming their prey. Predation relies on a contact-dependent Tad-like pilus (i.e. the Kil system) with a single major pilin (KilP) and several minor pilins (e.g. KilL, KilM, and KilN) [241]. Minor pilins assemble into four distinct KilLMN tips. These tips are essential for pilus extension and penetrating neighboring prey cells, with each tip having a different efficacy in killing different prey species [241]. Killing furthermore depends on the recruitment of an incomplete, needleless, type III secretion system (T3SS), underscoring the complex molecular machinery that supports the broad range of prey species on which *M. xanthus* can grow. When starved, cells transition to fruiting body development. This developmental process is only partially understood [242]. Starvation triggers the stringent response, leading to the RelA-mediated synthesis of (p)ppGpp. The stringent response is thought to promote the production of A- and C-signals [242, 243]. A-signal may function as a broad-range quorum-sensing signal that controls the onset of aggregation and C-signaling supports short-range interactions. Supported by the Mrp pathway, including the transcriptional activator of *fruA* (MrpC), C-signaling leads to the activation of FruA [244, 245]. Together, MrpC and FruA regulate hundreds of genes involved in aggregation and sporulation. Some genes are under the combinatorial control of both transcription factors, including, for example, the *dev* operon affecting sporulation [246]. In parallel to this regulatory cascade, the starvation-induced production of another second messenger, c-di-GMP, was shown to activate an NtrC-like activator (Nla24) leading to EPS production, which plays a role in fruiting body development as well [247]. Within the fruiting body, a small fraction of cells differentiates into myxospores. Spores are tightly bound together and likely disperse as a unit, giving rise to a next generation of swarming cells that prey together. The life cycle of *M. xanthus* differs from that of other soil microbes in that it effectively lacks a single-cell stage.

***Dictyostelium discoideum**. Dictyostelium discoideum* is part of the cellular slime molds, which are widely found in soils and known for their aggregative life cycle [248]. The aggregative life cycle consists of a growth and an aggregation stage: during growth, amoebal cells proliferate by phagocytizing bacteria [249], and, when starved, cells enter an aggregation process that leads to the formation of slugs, which can migrate toward light and heat [250] and eventually develop into fruiting bodies. Fruiting bodies consist of a cellulose-encased stalk and a spore-filled sorus [120]. The onset of aggregation depends on the cAMP-dependent protein kinase, PKA, which triggers the expression of aggregation-related genes [251]. Aggregation relies on cAMP signaling [252]. Cells sense cAMP in their environment through the G-protein coupled receptor (GPCR) cAR1 [253], and produce cAMP in response, using adenylate cyclase A (ACA) [254]. The produced cAMP is also secreted and thereby relayed to neighboring cells. cAMP production is transient: cAR1 becomes desensitized, ACA remains active only shortly, and cytosolic (RegA) and secreted phosphodiesterases (PDE1, encoded by *pdsA*) hydrolyze cAMP to restore basal concentrations [120]. Together, these processes give rise to traveling cAMP waves [255]. Cells migrate toward higher cAMP concentrations, producing aggregation streams that result in mounds. Besides cAMP signaling, the aggregation process also relies on adhesin factors (e.g. TgrB1/C1) [120]. Mounds develop into slugs that consist of two dominant cell types: prestalk and prespore cells [256, 257]. Prestalk cells occur in the anterior part of slugs (~20%) and are the organizers leading slug migration, while prespore cells occur in the posterior part (~80%) and follow. Communication between prespore and prestalk cells is thought to control the proportions of both cell types (e.g. DIF-1) [258]. During fruiting body development, prestalk and prespore cells differentiate into stalk and spore cells, respectively. The transition from mounds to slugs and then fruiting bodies in part depends on the consecutive activity of early and late promoters of *carA*, *acaA*, and *pdsA*, driving cAMP dynamics [259–261]. Over time, cAMP concentrations increase and activate distinct cAMP-sensing GPCRs (cAR1, cAR3, cAR2, cAR4) [262]. cAMP signaling also controls sporulation. By inactivating RegA, cAMP concentrations increase and mediate the PKA-dependent expression of sporulation genes [263]. RegA and PKA in other *Dictyostelia* species are also involved in encystation, leading to microcysts, suggesting that cAMP signaling is broadly involved in regulating dormancy [264, 265]. Besides the asexual life cycles leading to fruiting body development and microcysts, *Dictyostelia* can also undergo a sexual life cycle, where cells from opposite mating types undergo syngamy and generate a giant zygotic cell [266, 267]. The zygotic cell attracts and engulfs surrounding *Dictyostelia* cells, producing a cellulose-encased macrocyst. The molecular processes underlying macrocyst formation remain largely understudied.

***Aspergillus nidulans**. Aspergillus nidulans* is a model species within the *Ascomycota* and is part of the *Aspergillaceae* family that is abundantly found in soils. *Aspergillus* has a complex life cycle, with sexual, asexual, and parasexual reproduction [110, 188]. The ascospore gives rise to a haploid mycelium that is also called the homokaryon, consisting of pluripotent branching hyphae that grow apically. The mycelium can give rise to asexual spores—conidiospores—by developing spore-bearing structures called conidiophores. Conidiation is triggered by light and air exposure, besides other environmental cues, and begins with the formation of a foot cell from which a stalk emerges. The stalk cells give rise to vesicles, which undergo multipolar budding, producing metulae. The metulae subsequently give rise to phialides that support the formation of aerial spores, so-called conidia. The onset of conidiation depends on the light-responsive VeA complex [268, 269] and FluG, which is required for the production of an extracellular signaling factor. FluG signaling inhibits vegetative hyphal growth and promotes autolysis, putatively freeing up resources [270]. FluG is essential for conidiation and indirectly drives the activation of early regulatory factors, like Flb factors (e.g. FlbB) [110, 271]. These early factors are involved in vesicle swelling and metulae development and are followed by the activation of a regulatory backbone—consisting of BrlA, AbaA, and WetA—that supports phialide formation and conidiogenesis [272]. BrlA mediates the expression of conidiation-specific genes, including those underlying phialide production; AbaA regulates phialide differentiation; and WetA is required for pigmentation and conidial integrity [110]. Another factor downstream of BrlA, VosA, is essential for sporogenesis and spore maturation [273]. Besides producing conidiospores, haploid mycelia can also undergo sexual and parasexual reproduction [274, 275]. In the parasexual cycle, hyphal fusion produces a heterokaryon, within which haploid nuclei can fuse to produce diploid nuclei that can subsequently undergo haploidization. Sexual development can occur in either homokaryotic or heterokaryotic mycelia. Ascogenous hyphae form asci, within which nuclear fusion produces transient diploid nuclei that undergo meiosis, resulting in ascospores. The asci are contained within the cleistothecium, which promotes dispersal.

***Caenorhabditis elegans*** *.* Nematodes are among the most abundant metazoans in the soil, showing a broad range of feeding behaviors, with bacterial feeders, fungal feeders, and plant parasites. The best-studied nematode is *C. elegans*, which belongs to the broader class of *Chromadorea* that is abundantly observed in soils [276, 277]. In contrast to the microbial life cycles described above, the life cycle of *C. elegans* is largely deterministic [278, 279]. This determinism follows from development processes that are largely shielded from their environment: embryogenesis up to gastrulation occurs *in utero*; after egg laying, development continues *ex utero* for another 9 h before L1 larvae hatch [280]. L1 larvae consist of exactly 558 cells, including several hundred neurons and tens of muscle, pharynx, and intestine cells, among others [281]. The L1 larvae pass through four stages (L1–L4) before reaching their mature form. Under growth-promoting conditions, maturation requires 2 to 3 days. Under growth-limiting conditions, L1 larvae can enter the pre-dauer L2d stage and subsequently develop into dauer larvae [282]. Dauers arrest development and stop feeding, while forming a thick protective cuticle that covers the entire body and seals the mouth. Dauers are highly resistant to environmental stress and can survive for months under starvation, desiccation, and heat stress [283, 284]. Larvae enter diapause when exposed to nutrient-poor conditions, high population densities, or high temperatures. Population densities are sensed through the dauer pheromone consisting of ascarylose-derived glycolipids (ascarosides) [198]. The dauer pheromone alone can be sufficient to induce dauer formation. That is, when nutrient conditions are plentiful while concentrations of dauer pheromone are high, larvae can still enter diapause. The cues inducing dauer formation are processed through sensory neurons that ultimately affect the insulin/IGF-1 and TGF-β endocrine pathways [285, 286], reducing TGF-β and insulin signaling [282]. These pathways converge on steroid hormone signaling, reducing dafachronic acid (DA) levels, thereby switching the nuclear hormone receptor DAF-12 to its unliganded state and promoting dauer formation [287]. When conditions improve, insulin and TGF-β signaling increases, which increases DA levels and promotes the induction of normal

reproductive development. Dauers resume development by molting into an L4 larvae that proceeds to maturate. Apart from resisting environmental stress, dauers can also directly promote dispersal. Much like microbial fruiting bodies that protrude from the surface to promote dispersal, dauers can lift themselves into the air, either individually—through nictation—or collectively—through towering [118]. During nictation, a dauer larva lifts the anterior part of its body into the air while waving. The molecular underpinnings of nictation have partly been elucidated, showing an important role for a specific set of IL2 neurons [288]. In sum, even though *C. elegans* shows a largely deterministic life cycle, the life cycle motif with a facultative intermittent dormant life stage shows interesting commonalities with that of other soil microbes.

Box 2 Questions related to the life cycle ecology of soil microbes.

- How do microbial life cycles emerge in soil environments?
- What environmental conditions are microbes exposed to along their life cycle?
- How does the tempo of life cycle changes relate to that of environmental changes (e.g. wet–dry cycles)?
- What environmental conditions support life cycle closure (e.g. environmental scaffold)?
- What is the relevant spatiotemporal scale for understanding life cycle closure in soil environments?
- What ecological interactions support and/or hinder life cycle progression?
- How do ecological interactions change with life stages?
- How are growth and/or propagation dynamics coupled between species?
- How does life cycle coupling affect spatiotemporal species dynamics within soil communities?
- What life stages are most important for shaping community dynamics?
- How do life stages affect the long-term coexistence of microbial taxa in soils?

## Supplementary Material

Supplementary_Text_1_wrag215

## Data Availability

Not applicable as no data were purposely generated or analyzed for this review.
