# Supplementary material for: Lives in a gram of soil: linking life cycle biology to soil ecology
Source: ISME J. 2026 Aug 20;20(1):wrag215. doi: 10.1093/ismejo/wrag215 (PMC13657050; doi:10.1093/ismejo/wrag215)
Supplement: Supplementary file 1 [file supplementary_text_1_wrag215.pdf]

Supplementary Text 1. Footnotes to main text

Footnote 1. The terms ‘microbe’ or ‘microbial’ are used in an informal manner to refer to small organisms, without strictly discriminating between unicellular and multicellular organisms, subvisible and visible organisms, or prokaryotic and eukaryotic organisms.

Footnote 2. The life cycle is seen as the unit of selection [1] or, as John Tyler Bonner phrased it [2]: “The view taken here is that the life cycle is the central unit in biology. The notion of the organism is used in this sense, rather than that of an individual at a moment in time, such as the adult at maturity. Evolution then becomes the alternation of life cycles through time; genetics the inheritance mechanism between cycles, and development all the changes in structure that take place during the life cycle” [p. 3].

Footnote 3. This approach comes with two important challenges: (1) defining lineage tracking rules and (2) defining delineation criteria. Different tracking rules and delineation criteria can be compatible for describing a microbe’s life cycle, but those that best individuate reproducible life stages in time and space – as produced by their underlying developmental processes – are expected to be most informative in quantifying reproductive success [3–6].

Footnote 4. Life cycle properties related to the growth and propagation are more broadly described as life history traits and relate to for example the timing and rate of propagation, growth rate, longevity, lifetime reproductive success, trade-offs between growth and propagation and resource allocation [7].

Footnote 5. The convergence of developmental processes towards a canalized trajectory of phenotypic changes, i.e., a life cycle trajectory, as opposed to a single phenotypic state, is referred to as homeorhesis [8].

Footnote 6. Microbes can express so-called bet-hedging strategies, where phenotypic heterogeneity is an evolutionary adaptation in response to environmental fluctuations: by expressing distinct phenotypes, cells are adapted to distinct future environments, thereby increasing the reproductive output of a population when exposed to unexpected environmental changes. In a more formal sense, bet-hedging strategies maximize the geometric mean fitness, as determined by the multiplicative process of reproduction over time [9]. Bet-hedging strategies have been implicated in a wide range of microbial behaviors [10–12], but for most microbes conclusive evidence is lacking, because it remains difficult to map the reproductive fate of microbial cells within their natural environment [9].

Footnote 7. Expressing deterministic life cycle changes poses a significant developmental challenge. For instance, in the case of the aggregative life stages in *Dictyostelia*, co-aggregation of different strains can impair fruiting body development due to conflicts [13]. To assure reproducibility, developmental processes underlying aggregation should therefore somehow exclude defective cells that do not support fruiting body formation [14], much like – during normal multicellular development – defective cells (e.g., infected cells and cancerous cells) should be removed during growth as well. Several mechanisms can mediate such exclusion. For instance, in the case of *Dictyostelium discoideum*, strain-specific heterotypic adhesion proteins favor aggregation between cells from the same strain [15]. By excluding other strains, interference during aggregation is minimized. In general, developmental processes that canalize the reproducible expression of beneficial fruiting body properties are expected to be favored (when not too costly), whether they prevent other strains from co-aggregating or improves reproducibility otherwise [16] – e.g., robust aggregation across surface conditions.

Footnote 8. Genetic changes could support the diversification of an existing life cycle motif or result in new motifs, which are associated with novel life stages. For a novel life stage to emerge, species need to evolve emergent phenotypes that benefit either growth or propagation, overcoming constraints or trade-offs associated with the ancestral life cycle motif [17, 18], and coordinate the expression of these phenotypes in a reproducible manner [1]. Beneficial phenotypes might first be expressed sporadically, under specific conditions only, allowing cells to reap benefits occasionally. But as mechanisms evolve that improve, control and canalize their expression, beneficial phenotypes might become expressed in

a more and more coordinated manner, allowing for their reproducible appearance over time. This could ultimately lead to the establishment of a novel life stage. The evolutionary origin of a novel life stage is expected to go hand-in-hand with that of a novel taxonomic group: much like the origin of endosporulation gave rise to the *Firmicutes* and that of aggregative fruiting body development to *Dictyostelia*. As new disparate life cycles emerge and phylogenies expand over millions of years of evolution, soil communities will change as well. Since the origin of soils about 450 million years ago [19], with the colonization of land, communities have evolved to become the most diverse on the planet [20]; soils can therefore be seen as a hotpot of evolutionary innovation [21].

Footnote 9. Dynamical coupling is studied mathematically in the framework of dynamical systems theory, but it is also an important concept in ecology, for example, in studies that explore the coupling of population dynamics [22–25]. With the concept of life cycle coupling, I aim to highlight that dynamical coupling can occur in a life stage-dependent manner. For this essay, I mainly focus on the coupling of growth and/or propagation dynamics between microbes, but coupling can in principle also occur between microbial and mesoscopic or even macroscopic organisms (e.g., growth coupling between pathogens and their host).

References

1. van Gestel J, Tarnita CE. On the origin of biological construction, with a focus on multicellularity. *Proc Natl Acad Sci USA* 2017;**114**:11018–11026. https://doi.org/10.1073/pnas.1704631114

2. Bonner JT. Size and Cycle: An Essay on the Structure of Biology. Princeton University Press, 1965.

3. Griesemer J. Development, culture, and the units of inheritance. *Philos Sci* 2000;**67**:368.

4. van Gestel J, Wagner A. Cryptic surface-associated multicellularity emerges through cell adhesion and its regulation. *PLoS Biol* 2021;**19**:e3001250. https://doi.org/10.1371/journal.pbio.3001250

5. Bourrat P. Adding causality to the information-theoretic perspective on individuality. *Euro Jnl Phil Sci* 2024;**14**:9. https://doi.org/10.1007/s13194-023-00566-1

6. Fábregas-Tejeda A. The Organism-Environment Pairing: A Historical and Philosophical Reappraisal. MIT Press, 2026.

7. Frank SA. Microbial Life History: The Fundamental Forces of Biological Design. Princeton Oxford, 2022.

8. Waddington CH. The Strategy of the Genes: A Discussion of Some Aspects of Theoretical Biology. London: George Allen and Unwin, 1957.

9. Simons AM. Modes of response to environmental change and the elusive empirical evidence for bet hedging. *Proc Biol Sci* 2011;**278**:1601–1609. https://doi.org/10.1098/rspb.2011.0176

10. Solopova A et al. Bet-hedging during bacterial diauxic shift. *PNAS* 2014;**111**:7427–7432. https://doi.org/10.1073/pnas.1320063111

11. Hernandez-Valdes JA, van Gestel J, Kuipers OP. A riboswitch gives rise to multi-generational phenotypic heterogeneity in an auxotrophic bacterium. *Nat Commun* 2020;**11**:1203. https://doi.org/10.1038/s41467-020-15017-1

12. van Gestel J et al. *Bacillus subtilis* in defense mode: Switch-like adaptations to protistan predation. *Proc Natl Acad Sci USA* 2025;**122**:e2518989122. https://doi.org/10.1073/pnas.2518989122

13. Strassmann JE, Queller DC. Evolution of cooperation and control of cheating in a social microbe. *Proceedings of the National Academy of Sciences* 2011;**108**:10855–10862. https://doi.org/10.1073/pnas.1102451108

14. Ostrowski EA. Enforcing cooperation in the social amoebae. *Curr Biol* 2019;**29**:R474–R484. https://doi.org/10.1016/j.cub.2019.04.022

15. Hirose S et al. Self-recognition in social amoebae is mediated by allelic pairs of *tiger* genes. *Science* 2011;**333**:467–470. https://doi.org/10.1126/science.1203903

16. Clarke E. The multiple realizability of biological individuals. *The Journal of Philosophy* 2013;**110**:413–435.

17. Bourrat P et al. Tradeoff breaking as a model of evolutionary transitions in individuality and limits of the fitness-decoupling metaphor. *Elife* 2022;**11**:e73715. https://doi.org/10.7554/eLife.73715

18. Zhu M, Dai X. Shaping of microbial phenotypes by trade-offs. *Nat Commun* 2024;**15**:4238. https://doi.org/10.1038/s41467-024-48591-9

19. Lenton TM et al. First plants cooled the Ordovician. *Nature Geosci* 2012;**5**:86–89. https://doi.org/10.1038/ngeo1390

20. Anthony MA, Bender SF, van der Heijden MGA. Enumerating soil biodiversity. *Proc Natl Acad Sci USA* 2023;**120**:e2304663120. https://doi.org/10.1073/pnas.2304663120

21. Beaud Benyahia B et al. Terrabacteria: redefining bacterial envelope diversity, biogenesis and evolution. *Nat Rev Microbiol* 2025;**23**:41–56. https://doi.org/10.1038/s41579-024-01088-0

22. Allesina S, Tang S. Stability criteria for complex ecosystems. *Nature* 2012;**483**:205–208. https://doi.org/10.1038/nature10832

23. May RM. Will a large complex system be stable? *Nature* 1972;**238**:413–414. https://doi.org/10.1038/238413a0

24. Hastings A. Transient dynamics and persistence of ecological systems. *Ecol Lett* 2001;**4**:215–220. https://doi.org/10.1046/j.1461-0248.2001.00220.x

25. Hastings A et al. Transient phenomena in ecology. *Science* 2018;**361**:eaat6412. https://doi.org/10.1126/science.aat6412
